# Gastruloids as in vitro models of embryonic blood development with spatial and temporal resolution

**DOI:** 10.1038/s41598-022-17265-1

**Published:** 2022-08-04

**Authors:** Giuliana Rossi, Sonja Giger, Tania Hübscher, Matthias P. Lutolf

**Affiliations:** 1 Laboratory of Stem Cell Bioengineering, Institute of Bioengineering, School of Life Sciences and School of Engineering, École Polytechnique Fédérale de Lausanne (EPFL), Vaud, 1015 Lausanne, Switzerland; 2 Institute of Chemical Sciences and Engineering, School of Basic Science, École Polytechnique Fédérale de Lausanne (EPFL), Vaud, 1015 Lausanne, Switzerland; 3 Present Address: Roche Institute for Translational Bioengineering (ITB), Roche Pharma Research and Early Development, Roche Innovation Center Basel, Basel, Switzerland

**Keywords:** Biological models, Embryogenesis, Haematopoiesis

## Abstract

Gastruloids are three-dimensional embryonic organoids that reproduce key features of early mammalian development in vitro with unique scalability, accessibility, and spatiotemporal similarity to real embryos. Recently, we adapted the gastruloid culture conditions to promote cardiovascular development. In this work, we extended these conditions to capture features of embryonic blood development through a combination of immunophenotyping, detailed transcriptomics analysis, and identification of blood stem/progenitor cell potency. We uncovered the emergence of blood progenitor and erythroid-like cell populations in late gastruloids and showed the multipotent clonogenic capacity of these cells, both in vitro and after transplantation into irradiated mice. We also identified the spatial localization near a vessel-like plexus in the anterior portion of gastruloids with similarities to the emergence of blood stem cells in the mouse embryo. These results highlight the potential and applicability of gastruloids to the in vitro study of complex processes in embryonic blood development with spatiotemporal fidelity.

## Introduction

Mammalian embryos develop in the uterus and are dependent on maternal interactions, which raises scientific and ethical challenges in accessing them for developmental studies. Embryonic organoids are 3D models that are experimental alternatives to mammalian embryos and offer the unprecedented potential to study aspects of embryogenesis in vitro. Due to their accessibility, scalability, and experimental versatility, embryonic organoids offer promising alternatives and complements to studies in animal models^1–7^. Gastruloids, a type of embryonic organoid, are aggregates of embryonic stem cells (ESCs) that mimic aspects of post-implantation development, such as symmetry breaking, gastrulation and establishment of the three major body axes, when cultured under the correct conditions^6,8^.

Recently, we have shown that gastruloid culture conditions can be steered to promote early cardiovascular development, or the formation of what resembles a vascular network, and a cardiac primordium^9^. Cardiovascular development is connected with blood emergence and early blood development depends on the endothelial-to-hematopoietic transition (EHT), a process in which vascular cells of the hemogenic endothelium progressively lose their endothelial signature and activate a hematopoietic transcriptional program^10–12^. The development of the hematopoietic system occurs in two successive, spatially and temporally restricted waves^13^. Primitive hematopoiesis begins around embryonic day 7.5 (E7.5) in the yolk sac blood islands, and is defined by the initial wave of blood cell production before circulation is established^14,15^. After the establishment of circulation, definitive hematopoiesis takes place from E8.5 to E10.5 at various embryonic sites: the placenta, the aorta-gonad-mesonephros (AGM) region, and the umbilical and vitelline arteries^16–19^. During later embryonic development, the fetal liver is colonized through circulation, and becomes the main organ for hematopoietic stem cell (HSC) expansion and maturation^20,21^. Around E16.5, hematopoietic progenitors from the fetal liver start to colonize the developing bones to form the classical adult hematopoietic stem cell niche^22,23^. Due to the endothelial origin of blood cells, we hypothesized that we could capture the early stages of blood development in gastruloids.

Here we show that gastruloids cultured in cardiovascular-inducing conditions^9^ display a hematopoiesis-related transcriptional signature and express surface markers characteristic of early hematopoietic cells. Specifically, we describe the emergence of a population of cells with CD34, c-Kit, and CD41 markers, which correspond to an early blood progenitor phenotype. We also describe a population of Ter-119^+^ cells that display an erythroid-like phenotype. Analogously to embryonic development, we observed that blood progenitors accumulate from 96 to 168 h in gastruloids and, at 168 h, we observed their multilineage clonogenic potential both in vitro and in vivo. We also discovered that these cells are located anteriorly, in proximity to the vascular-like plexus, in line with observations in mammalian embryos. Taken together, these data suggest that gastruloids can model the early stages of hematopoietic development and have great utility as an in vitro model system to study mechanisms of blood development.

## Results

### Gastruloids display a hematopoiesis-related transcriptional signature

**Figure 1:**
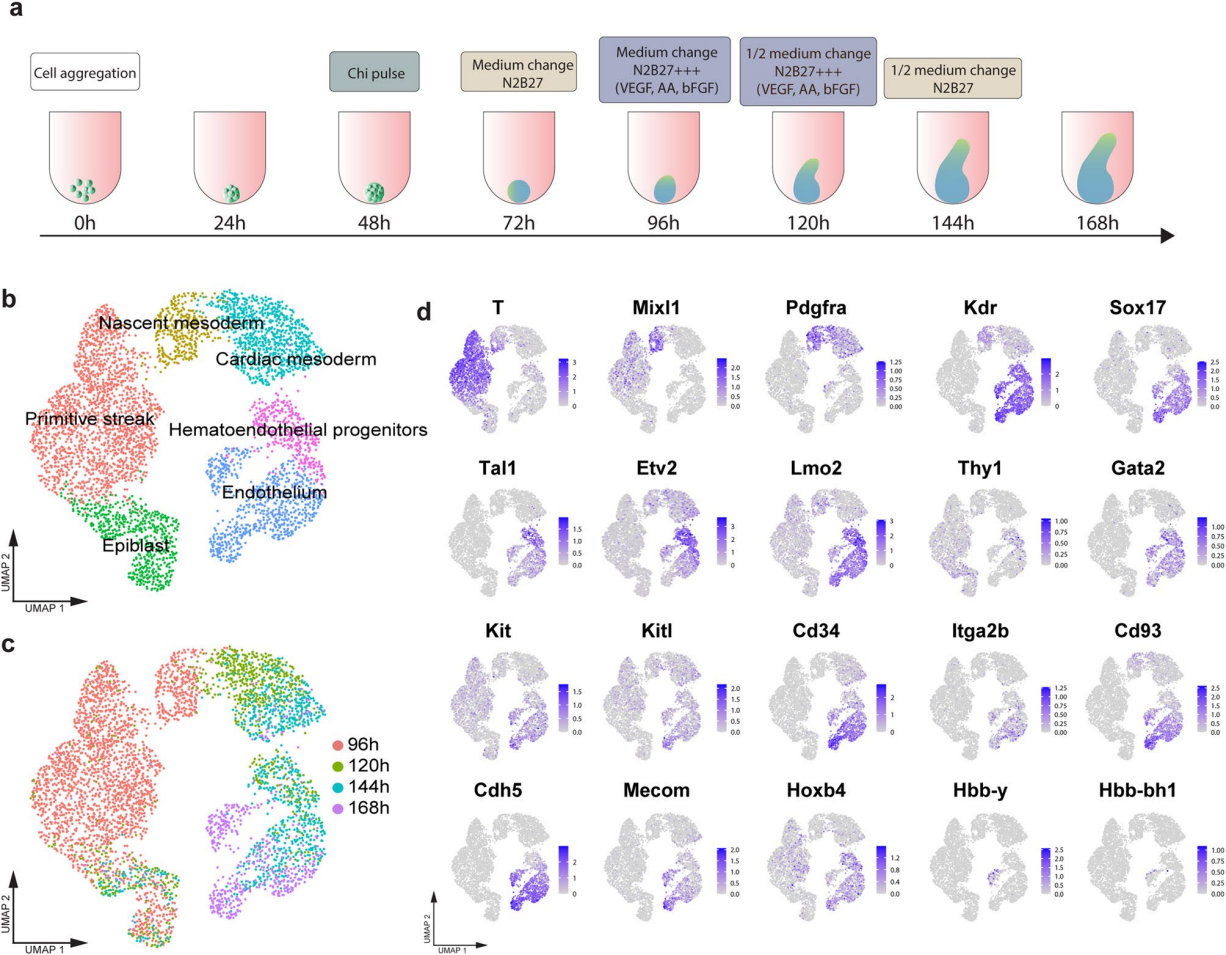
Gastruloids express markers of early blood development. (**a**) Experimental outline of the protocol used for gastruloid generation. (**b–c**) UMAP plots showing (**b**) clusters on the trajectories from epiblast to endothelium and (**c**) the distribution of cells in the UMAP plots according to analyzed time points. (**d**) UMAP plots showing the expression of hematopoiesis-related genes in the scRNA-seq dataset. n= 2 replicates per time point. Dataset from^9^.

We recently described a protocol to promote cardiovascular development in gastruloids, which required the addition of VEGF, bFGF, and ascorbic acid (AA) to standard gastruloid growth culture conditions^9^ (Fig. 1a). As VEGF and bFGF are necessary for hematopoiesis in vitro and in vivo^24–30^, we hypothesized that these culture conditions could also support the development of the blood lineage. Thus, we mined the scRNA-seq dataset of gastruloids grown in the presence of VEGF and bFGF^9^, with a focus on clusters that were on the trajectory from epiblast to endothelial progenitors (Fig. 1b). From gene expression analysis of cells in these clusters, we noted the progressive emergence of a population that expressed characteristic markers of blood progenitors. Around 96 h (Fig. 1c), we observed a population of cells expressing *T/Brachyury*, *Mixl1*, *Pdgfra,* and *Kdr/Flk1/Vegfr2*, which was indicative of mesoderm patterned to a hematopoietic fate^31^ (Fig. 1d). Co-expression of *Kdr* and *T*/*Brachyury* is known to mark common progenitors for cardiac, endothelial, and hematopoietic lineages^32,33^. We also noted that *Sox17*, which marks the hemogenic endothelium in vivo^34^, was upregulated over time, along with transcription factors and surface antigens typically associated with hematopoietic development, such as *Tal1/SCL*, *Etv2*, *Lmo2*, *Thy1*, and *Gata2* (Fig. 1d)^35–39^. In later stages of development, around 144 h to 168 h (Fig. 1c), we observed the start of the expression of *Kit*(c-Kit) and its ligand *Kitl*, *Cd34*, *Itga2b/Cd41*, *Cd93*, *Cdh5/VE-cadherin*, and *Mecom/Evi-1*, which are typical markers of developing blood progenitors^12,27,40–45^ (Fig. 1d). In line with previously reported observations^8^, the gastruloids also expressed *Hoxb4* (Fig. 1d), which has been shown to promote hematopoietic differentiation upon ectopic expression in vitro^46,47^*.* Finally, at 168 h, we observed a small population that expressed the hemoglobin embryonic isoforms *Hbb-y* and *Hbb-bh1*^48^ (Fig. 1d). Intriguingly, the hemoglobin genes were associated with an endothelial sub-cluster that expressed typical endocardial genes^9^, which suggests their emergence from hemogenic endocardium^49^. We performed RT-qPCR analysis of gastruloids at different time points, which confirmed the progressive upregulation of hematopoiesis-related factors (Fig. S1a–e), as well as the expression of *Hoxb4* (Fig. S1f). We also observed the expression of embryonic (*Hbb-y*, *Hbb-bh, Hba-x*) but not adult (*Hbb-b1*, *Hbb-b2*, *Hba-a1*) hemoglobin isoforms (Fig. S1g–l). Taken together, these results suggest that gastruloids express key hematopoiesis-related genes in a temporal sequence that follows embryonic development.

### Early blood cells in gastruloids are identified by surface marker analysis

We can examine the expression of typical surface markers to identify blood progenitors. Towards this goal, we performed flow cytometry analysis of gastruloids from 96 to 168 h to determine the expression of canonical early hematopoietic markers across three different cell lines: *Sox1-GFP::Brachyury-mCherry*^50^, *Flk1-GFP*^51^, and *Gata6-Venus*^52^. We first observed the expression of the hematopoietic markers CD34, c-Kit, and CD93. As a marker of early expression during embryonic development, CD34 was upregulated from 120 h (Fig. S2a). CD34 marks both vascular and hematopoietic progenitors within the hemogenic endothelium^53^. c-Kit was expressed with some fluctuations throughout the gastruloid culture (Fig. S2b). While both c-Kit and CD34 are consistently expressed in early embryonic hematopoietic cells as well as adult HSCs, their expression is not restricted to these cell types during development^40,44,45,53,54^. We noted that CD93 was upregulated before 120 h, and its levels increased progressively over time, most evidently in the *Sox1-GFP::Brachyury-mCherry* and *Gata6-Venus* cell lines (Fig. S2c). CD93 is expressed early on during embryonic development, in both endothelial cells and hematopoietic progenitors^55^.

Between 144 and 168 h, we then observed an accumulation of CD41^+^ cells, which is a key marker for the onset of hematopoiesis in the embryo^27,42^; this accumulation was most prominent in the *Gata6-Venus* cell line (Fig. S2d). Of note, it was previously shown that early hematopoietic progenitors lack the expression of the pan-hematopoietic markers CD45 and Sca1, which are upregulated upon later stages of blood ontogeny^22^. In line with these in vivo findings, CD45^+^ and Sca1^+^ cells emerged only during later stages of gastruloid development (Fig. S2e,f). Additionally, we looked for the expression of Ter119, an erythroid lineage marker that is upregulated in the embryo from E8.0 in evolving erythroid progenitor cells, with concomitant downregulation of the primitive hematopoietic marker CD41^42^. Ter119^+^ cells emerged in gastruloids around 120 h and were maintained on a similar level until 168 h (Fig. S2g). Finally, the endothelial markers CD31 and Flk1 were also upregulated starting around 120 h (Fig. S2h,i), which correlates with previous reports^9^.

**Figure 2:**
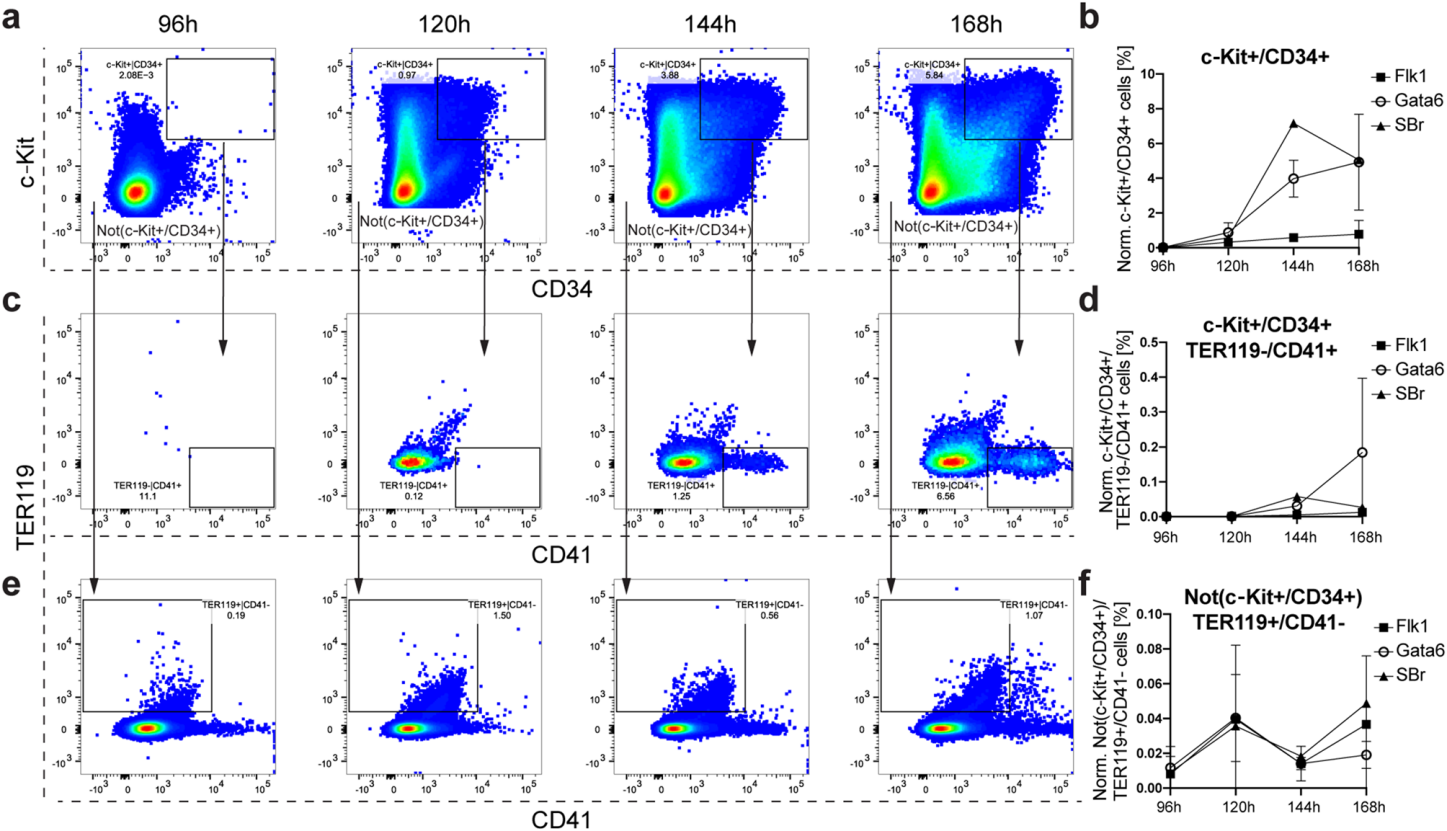
Emergence of hematopoietic and erythroid progenitors in gastruloids. (**a**) Representative flow cytometry plots and (**b**) relative quantification of the emergence of the c-Kit^+^/CD34^+^ populations in gastruloids from 96 to 168 h. The c-Kit^+^/CD34^+^ population was gated further for TER119^−^/CD41^+^ populations, with (**c**) representative flow cytometry plots and (**d**) relative quantification. We refer to the remaining cells after gating for c-Kit^+^/CD34^+^ cells as Not(c-Kit^+^/CD34^+^). Notably, the Not(c-Kit^+^/CD34^+^) population still contains single positive cells for c-Kit and CD34. The Not(c-Kit^+^/CD34^+^) population was additionally gated for TER119^+^/CD41^−^, with (**e**) representative flow cytometry plots and (**f**) relative quantification. The graphs in (**b**, **d**, **f**) show the changes in the percentage of positive cells over time. *Flk1-GFP* (Flk1) (n = 2 replicates), *Gata6-Venus* (Gata6) (n = 2 replicates) and *Sox1-GFP::Brachyury-mCherry* (SBr) (n = 1 replicate). Data are represented as mean ± SD.

The concomitant upregulation of hematopoietic and vascular surface markers in gastruloids suggests the emergence of hematoendothelial progenitors. Although such markers are not unique to hematopoietic cells, combinations of markers can define them. A published assessment of the colony formation potential during early embryonic stages (from E7.0 to E9.5) showed that CD34^+^/c-Kit^+^/CD41^+^ cells from the yolk sac, AGM, and fetal liver held most of the multipotent capacity^42^. Interestingly, we found a consistent upregulation of the c-Kit^+^/CD34^+^ population in gastruloids from 120 h (Fig. 2a,b), whereas the c-Kit^+^/CD34^+^/TER119^−^/CD41^+^ population only appeared after 144 h (Fig. 2c,d).

The emergence of erythroid cells in the embryo occurs from E8.0 onwards and is marked by the upregulation of the erythroid lineage marker Ter119 with subsequent downregulation of CD41^42^. We observed that Not(c-Kit^+^/CD34^+^)/ Ter119^+^/CD41^−^ population is upregulated early on and fluctuated over the time course of the gastruloid culture (Fig. 2e,f). Our results suggest that primitive hematopoietic precursors and erythroid progenitor cells emerge during later stages of gastruloid development.

### CD41^+^ cells in gastruloids localize near a vascular-like network

**Figure 3:**
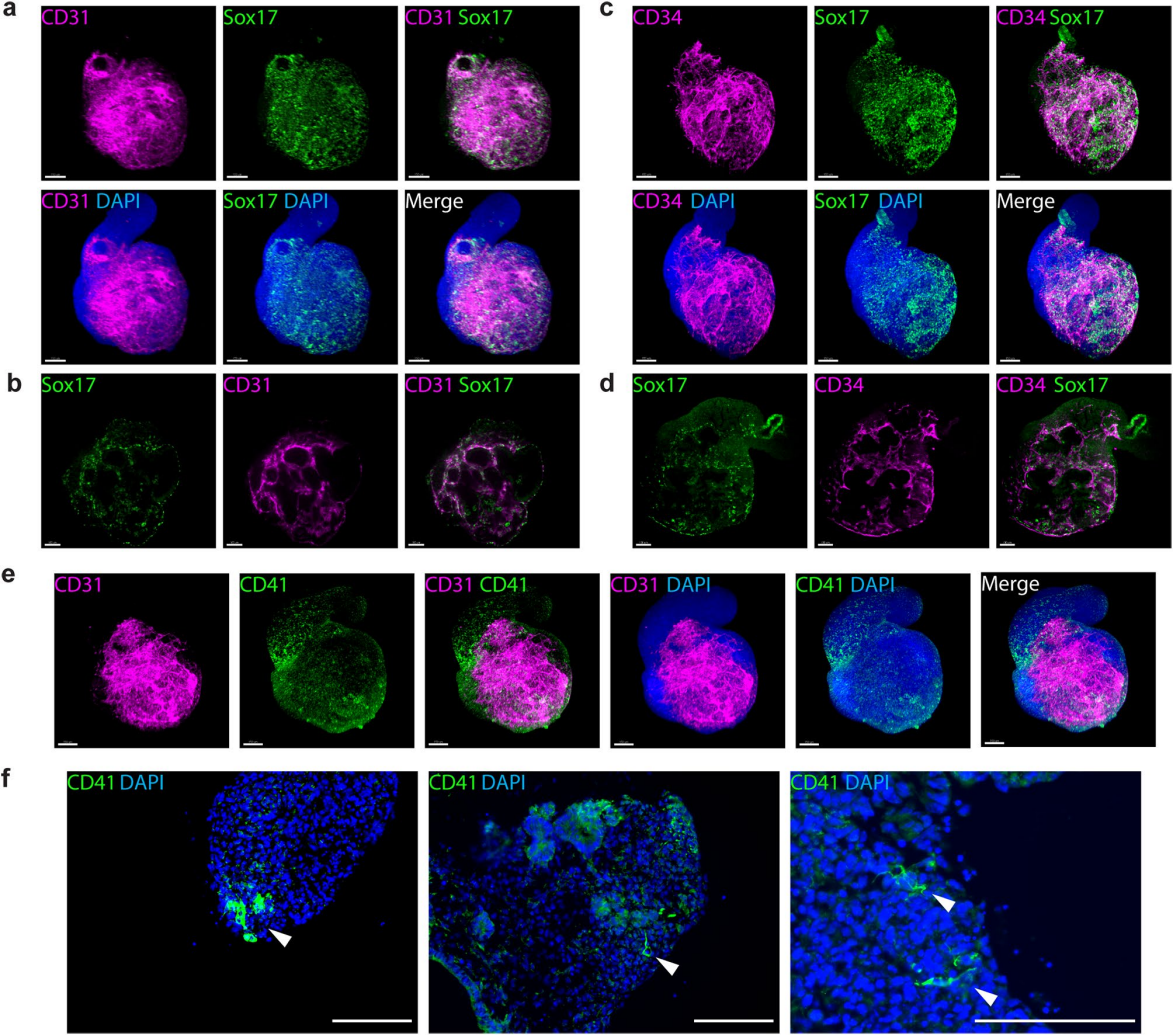
Emergence of blood progenitors in gastruloids. (**a**) Representative light-sheet images of *Gata6-Venus* gastruloids at 168 h showing the co-expression of CD31 and Sox17 in a 3D reconstruction and (**b**) in a single Z-plane. (**c**) Representative light-sheet images of *Gata6-Venus* gastruloids at 168 h showing the co-expression of CD34 and Sox17 in a 3D reconstruction and (**d**) a single Z-plane. (**e**) Representative light-sheet images of *Gata6-Venus* gastruloids at 168 h showing CD41^+^ clusters that arise near the CD31^+^ vascular network. (**f**) Representative sections of *Gata6-Venus* gastruloids at 168 h show CD41^+^ cells at higher magnification. Scale bars,100 µm.

Gastruloids display a highly coordinated temporal recapitulation of in vivo development and, most importantly, mimic the embryonic organization of tissues in space^8,9^. During development, CD41^+^ hematopoietic precursors are found near the Flk1^+^ endothelial network in the most proximal portion of the yolk sac^42^. Subsequently, the hemogenic endothelium is found in the AGM region located in the anterior portion of the embryo^56^. Within the hemogenic endothelium, Sox17 has been shown to play a functional role in HSC development and to mark hemogenic endothelial cells and emerging hematopoietic cells^34,57^. We discovered that Sox17 is expressed in the nuclei of CD31^+^ and CD34^+^ cells, which mark the vascular-like network, in the anterior region of gastruloids at 168 h (Fig. 3a–d and Fig. S3a,b). Moreover, we observed the expression of CD41 in clusters of cells located near the endothelial network that expressed CD31 (Fig. 3e,f and Fig. S3c). Taken together, these data suggest that gastruloids support the formation of hemogenic endothelial cells, from which CD41^+^ hematopoietic precursors emerge.

### Blood progenitors display multilineage clonogenic potential in vitro and in vivo

**Figure 4:**
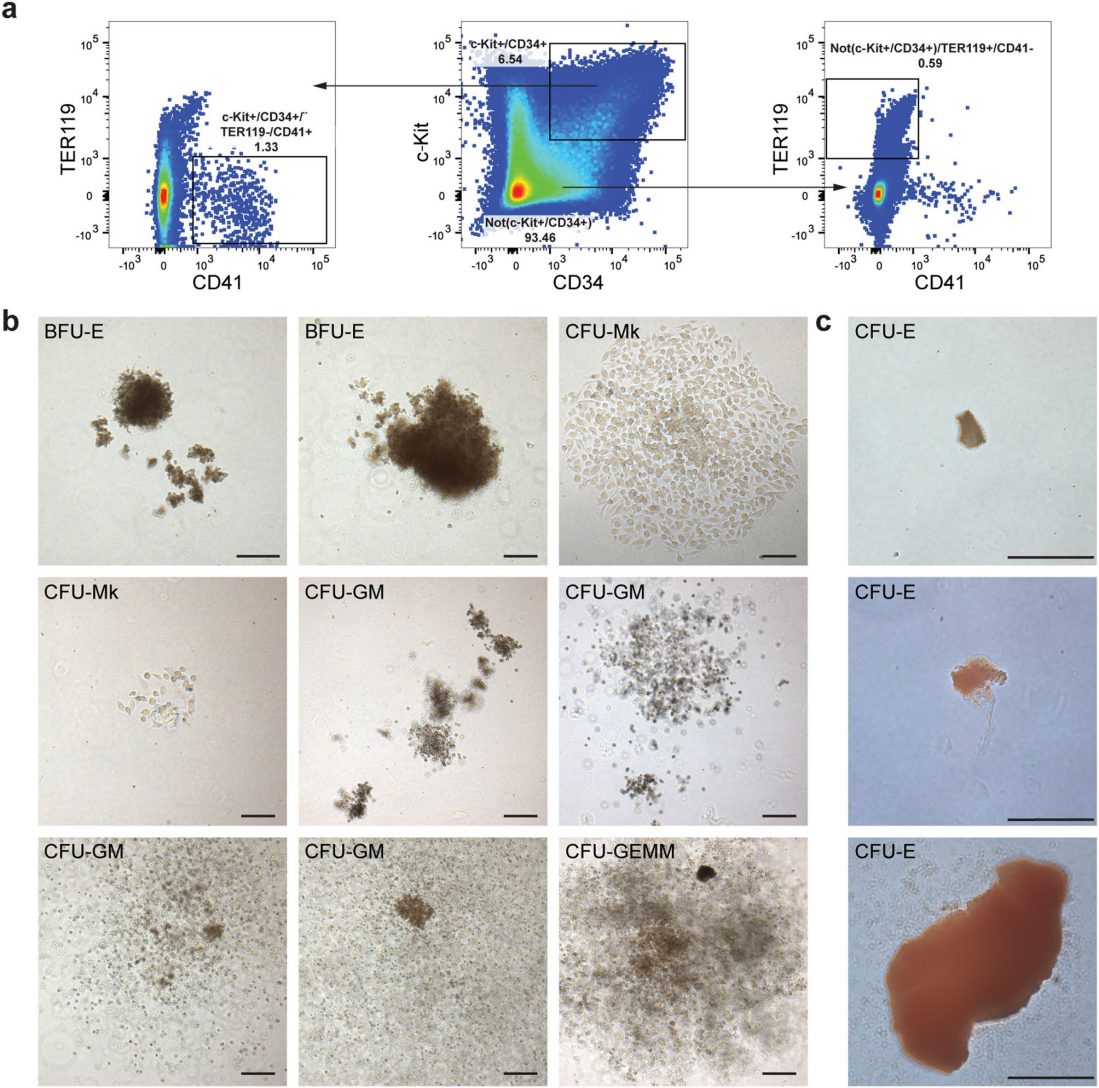
Multilineage clonogenic potential of gastruloid-derived blood progenitors. (**a**) Gating strategy for the isolation of c-Kit^+^/CD34^+^/TER119^−^/CD41^+^ hematopoietic progenitors and Not(c-Kit^+^/CD34^+^)/TER119^+^/CD41^−^ primitive erythroid cells from *Gata6-Venus* gastruloids at 168 h. (**b**) Representative images of BFU-E, CFU-Mk, CFU-GM, CFU-E and CFU-GEMM colonies derived from c-Kit^+^/CD34^+^/TER119^−^/CD41^+^ cells after 17 days of culture in Methocult. (**c**) Representative images of CFU-E colonies derived from the Not(c-Kit^+^/CD34^+^)/TER119^+^/CD41^−^ population after 17 days of culture in Methocult. *BFU-E* burst-forming-unit-erythroid, *CFU-Mk* colony forming unit-megakaryocyte, *CFU-GM* colony forming unit-granulocyte, macrophage, *CFU-GEMM* colony forming unit-granulocyte, erythroid, macrophage, megakaryocyte, *CFU-E* colony forming unit-erythroid. In this Figure, we refer to the remaining cells after gating for c-Kit^+^/CD34^+^ cells as Not(c-Kit^+^/CD34^+^). Notably, the Not(c-Kit^+^/CD34^+^) population still contains single positive cells for c-Kit and CD34. Scale bars 200 μm.

To assess the multipotency of the blood progenitors that arise in gastruloids around 168 h, we isolated two potential populations by flow cytometry: a hematopoietic progenitor population marked by c-Kit^+^/CD34^+^/TER119^−^/CD41^+^ and a primitive erythroid population marked by Not(c-Kit^+^/CD34^+^)/TER119^+^/CD41^−^ (Fig. 4a). We tested both populations in a colony–forming-unit (CFU) assay. Notably, we observed the formation of morphologically different colonies derived from the c-Kit^+^/CD34^+^/TER119^−^/CD41^+^ hematopoietic progenitor population (Fig. 4b). Colonies were identifiable after 7 days from cell seeding and expanded from day 7 to day 14 (Fig. S4a), which suggested that the hematopoietic clones could form efficiently proliferating colonies. Specifically, we observed the formation of colonies that contained single, round-to-slightly elongated cells, which we classified as CFU-megakaryocyte (CFU-Mk); colonies with the typical appearance of CFU-granulocyte, macrophage (CFU-GM), which contained multiple clusters with a dense core of oval-to-round cells with a grey appearance (macrophages) or small, bright and round cells (granulocytes); potential CFU-granulocyte (CFU-G) colonies; and also multipotential-like colonies of CFU-granulocyte, erythroid, macrophage, megakaryocyte (CFU-GEMM), which contained multiple cell types from different lineages (Fig. 4b). Moreover, we noted the appearance of red-to-brown colonies that indicated the presence of erythroid progenitors, which contained individual erythroid clusters with tiny, irregularly shaped, and often fused cells typical of burst-forming-unit-erythroid (BFU-E) (Fig. 4b). The Not(c-Kit^+^/CD34^+^)/TER119^+^/CD41^−^ mainly gave rise to CFU-erythroid (CFU-E) colonies, which were mostly small and contained clusters of irregularly shaped and fused erythroblasts with a similar red-brown color to BFU-E colonies (Fig. 4c). These colonies were much smaller and did not show evident expansion from 7 to 14 days (Fig. S4b). Controls with c-Kit^+^/CD34^+^/TER119^−^/CD41^−^ and Not(c-Kit^+^/CD34^+^)/TER119^−^/CD41^−^ populations did not show any evident CFU potential and undifferentiated mouse embryonic stem cells (mESCs) formed only fibroblastic-like clusters (Fig. S4c). These data support the idea that the expression of CD41 is essential for the establishment of multipotent hematopoietic progenitor cells in gastruloids, akin to in vivo observations^41,42^.

**Figure 5:**
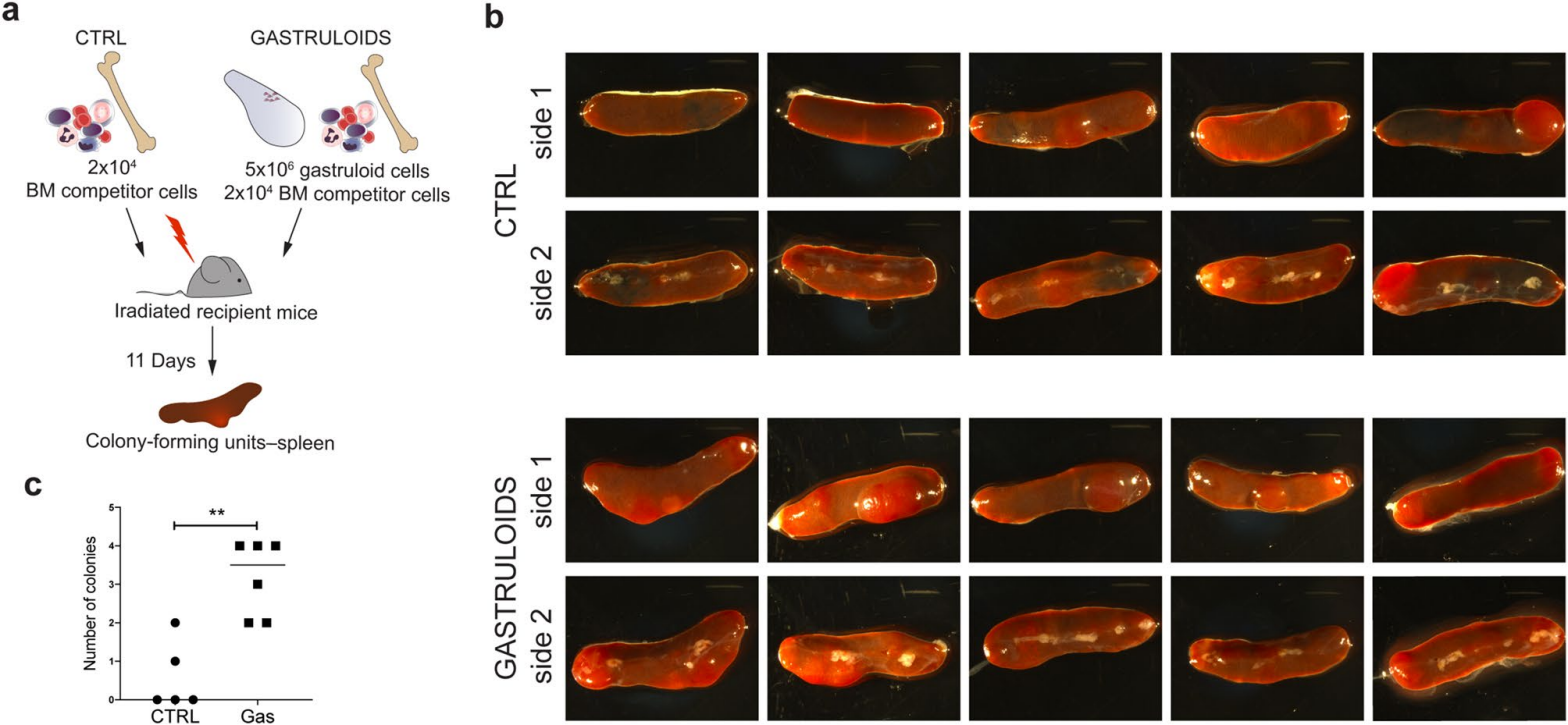
Gastruloid-derived cells form CFU-S upon transplantation. (**a**) Schematic overview of the transplantation strategy and analysis of CFU-S. (**b**) Stereomicroscope images of spleens from 5 different recipient mice imaged on both sides. (**c**) Quantification of the number of colonies observed in recipient mice transplanted with control cells or cells derived from *Gata6-Venus* gastruloids. Single data points are shown in graph. n = 5 control mice and n = 6 mice were injected with *Gata6-Venus* gastruloid cells. *CTRL* control, *BM* bone marrow.

Transplantation studies have shown that long-term repopulating HSCs (LTR-HSCs) appear after E10 to E11 in the AGM region of the embryo, a time point that is not attained with current gastruloid culture protocols. However, definitive colony-forming-unit-spleen (CFU-S) can been observed from E9.5^18,19,56^. We hypothesized that hematopoietic progenitors within gastruloids at 168 h harbored the potential to generate CFU-S upon transplantation in irradiated animals. As shown in Fig. 5, we dissociated the gastruloids into single cells at 168 h and injected 5 × 10^6^ gastruloid cells into the tail vein of lethally irradiated recipient mice together with 2 × 10^4^ competitor bone marrow cells. After 11 days, we assayed their capacity to form CFU-S relative to control mice, which were injected with only the competitor bone marrow cells (Fig. 5a). The presence of CFU-S was significantly higher in the spleens of recipient mice injected with gastruloid cells compared to control mice (Fig. 5b,c). Altogether, our data indicate that an hematopoietic progenitor population is developing in gastruloids at 168 h. Surface marker characteristics and the ability to form CFU-S upon in vivo transplantation suggest that this population is reminiscent of hematopoietic progenitors that arise between E8.5 and E9.5 in embryonic development.

## Discussion

The challenge of studying the development of the hematopoietic system highlights the need for relevant in vitro models that can recapitulate in vivo processes with spatial and temporal fidelity. We have recently shown that progenitor cells generated during gastruloid development are highly similar to their in vivo counterparts^9^, which is likely due to their recapitulation of developmental trajectories and their spatiotemporal response to mechano-chemical signals from proximal tissues. In this work, we showed that gastruloids expressed key hematopoietic genes in a temporally relevant fashion. A striking observation was that the gastruloids spontaneously expressed *Hoxb4* in the hematoendothelial and endothelial clusters. This finding supports previous works where ectopic expression of human *HOXB4* was shown to induce the expression of transcription factors necessary for hematopoiesis^46,47^.

Gastruloids also implement collinear *Hox* transcriptional patterns along the anteroposterior axis^8^ and form morphologically relevant organ primordia, in a similar fashion to the spatial organization of tissues in embryos^9^. Furthermore, numerous in vitro and in vivo experiments have previously revealed that, within the hemogenic endothelium, a Flk1^+^ precursor population has the ability to undergo endothelial-to-hematopoietic transition^10–12^. Such *Flk1*^+^ precursors were also detected in gastruloids, both by scRNA-seq and flow cytometry analysis, together with the expression of other canonical hematoendothelial markers.

We have previously shown that gastruloids develop an endodermal primitive gut tube-like structure^58^, which forms close to the cardiac compartment^9^. It is known that endoderm-derived factors are needed to guide hemogenic fate and HSC development^29,59–63^. We hypothesized that factors secreted by the endodermal compartment in gastruloids influence the development and localization of hemogenic cells. Indeed, we have described the emergence of CD41^+^ blood progenitor cells that arise in the anterior portion of gastruloids, in close proximity to the vascular-like network and the endodermal compartment. Furthermore, these evolving hematopoietic progenitor cells display multipotent clonogenic potential in CFU assays in vitro at later stages of gastruloid development, and showed CFU-S potential in vivo. Importantly, we observed the emergence of erythroid cells that are the first differentiated hematopoietic cells to appear in gastruloids, similar to what happens during embryonic development^13^. We have confirmed this by the expression of embryonic hemoglobin isoforms in gastruloids at 168 h. Intriguingly, we observe the expression of these genes within the endothelial cluster, and mainly in a sub-cluster that we previously associated with endocardial development^9^. This suggests that these cells might arise from the hemogenic endocardium, which is a known site for hematopoiesis at the early heart tube developmental stage^49^.

In addition to genetic and morphological studies, we compared different cell lines through flow cytometry analysis to corroborate our findings. We observed a similar trend in the expression of hematopoietic surface markers across distinct cell lines at different time points. However, we observed differences in the propensity of each cell line to generate cells that expressed typical blood progenitor markers. Our observations are in line with previous studies that suggest that the genetic background of the used cell line can skew mESC differentiation^64^ and gastruloid cell-type diversity^65^.

In conclusion, our data suggest that gastruloids are excellent candidates to model hematopoietic development in vitro in an embryo-like context. Gastruloids can serve as model systems to study the emergence of blood progenitors from different embryonic locations, to recapitulate key signals and developmental events, all with spatiotemporal fidelity.

In the future, gastruloids can be used to model the emergence of hematopoietic precursor cells with the potential to decipher their cellular origins and the complex interactions of blood precursors with the proximal endothelium. The simple scalability and facile manipulability of gastruloids provides the potential for large-scale production of hematopoietic progenitors in an embryo-like context. Since human PSC-derived gastruloids have recently been described^66^, future studies are likely to transfer this know-how to human models and open new avenues for human in vitro developmental studies, screening, and personalized medicine approaches. Indeed, previous attempts to generate HSCs in culture were mainly based on the use of embryoid bodies (EBs), through ectopic expression of key hematopoietic genes^47,67^, or a combination of morphogen-driven directed differentiation and transcription factor-mediated cell fate conversion^30,68^. Although these protocols have shown that transplantable cells can be generated, they are not ideal for capturing early developmental events with spatiotemporal fidelity. Moreover, ectopic expression of key hematopoietic genes represents a developmental shortcut that could bypass crucial steps in HSC development. Our developmental biology-inspired approach may instead lead to the generation of cells and progenitors with improved therapeutic relevance in the future. Finally, we envision that gastruloids have applicability beyond studies of cardiovascular and hematopoietic development and serve as in vitro models for other mechanistic studies of embryonic developmental biology.

## Material and methods

### Cell culture

mESCs were cultured as previously described^9^. Briefly, all cells were cultured at 37 °C, 5% CO_2_ in growth medium (DMEM, 10% Embryonic Stem Cell qualified FBS (Gibco), NEAA, Sodium Pyruvate, β-mercaptoethanol, 3 μM CHIR99021 (Chi), 1 μM PD025901 and 0.1 μg ml^−1^ LIF). *Gata6-Venus*^52^ and *Flk1-GF*P^51^ cells were cultured on gelatinized tissue-culture flasks; *Sox1-GFP::Brachyury-mCherry* cells^50^ without any coating. All cells were routinely tested for Mycoplasma with Mycoalert plus mycoplasma detection kit (Lonza) or by PCR.

### Gastruloid culture

Gastruloids were generated as previously described^6,9,69^. Briefly, 300–700 mESCs were aggregated in 40 μl N2B27 in 96-well Clear Round Bottom Ultra-Low Attachment Microplates (7007, Corning). After 48 h, 150 μl per well of 3 μM Chi in N2B27 were added. At 72 h, 150 μl of medium were removed and substituted with 150 μl of fresh N2B27. From 96 h, the medium was changed to N2B27+++ which contains 30 ng ml^−1^ bFGF (PMG0034, Gibco), 5 ng ml^−1^ VEGF 165 (PHC9394, Gibco) and 0.5 mM L-ascorbic acid phosphate (013-12061, Wako). From 120 h on, half of the medium was changed daily. From 144 h, N2B27 was used for daily medium changes. For immunofluorescence analysis, gastruloids at 96 h were transferred in Ultra-Low Attachment 24-Well Plates (3473, Corning) with 100 μl of medium, plus 700 μl of fresh N2B27+++, and cultured on an orbital shaker placed at 37 °C, 5% CO_2_ at 100 rpm (VWR mini shaker), with the same culture schedule.

### Whole mount immunofluorescence and light-sheet imaging

Immunofluorescence on whole-mount gastruloids was performed as previously described^9,69^. Briefly, gastruloids were washed with PBS and fixed in 4% PFA for 2 h at 4 °C on a rotating shaker. Samples were repeatedly washed with PBS and blocking buffer (PBS, 10% FBS, 0,2% Triton X-100), then blocked for 1 h at 4 °C with blocking buffer. Gastruloids were then incubated O/N with primary antibodies in blocking buffer, at 4 °C on a rotating shaker. On the following day, gastruloids were repeatedly washed with blocking buffer, at 4 °C while shaking, and incubated O/N with secondary antibodies and DAPI (2 μg ml^−1^, Sigma-Aldrich) in PBS, at 4 °C on a rotating shaker. On the following day, gastruloids were washed for 1 h with blocking buffer, at 4 °C on a rotating shaker, and rinsed in PBS, 0,2% FBS, 0,2% Triton X-100. For light-sheet imaging, gastruloids were mounted in 1% low melt agarose in glass capillaries, and cleared with CUBIC-mount solution^70^ O/N. CUBIC-mount was prepared as previously described^70^, by mixing 50% w/v sucrose, 25% w/v urea, and 25% w/v N,N,N′,N′-tetrakis(2-hydroxypropyl)ethylenediamine (Sigma-Aldrich) in distilled water, adjusted to a final refractive index of n = 1.45. Imaging was performed in CUBIC-mount solution with a Zeiss Light-sheet Z1 microscope equipped with a Plan-Neofluar 20 ×/1.0 Corr nd = 1.45 objective. Images were processed using Imaris software for 3D image analysis.

The following primary antibodies were used: rabbit anti-CD41 (ThermoFisher Scientific, PA5-79,526, 1:200); rabbit anti-CD34 (clone EP373Y, Abcam, 1:100); goat anti-Sox17 (R&D, AF1924, 1:200); rat anti CD31 (BD, MEC13.3, 1:100). The following secondary antibodies were used: donkey anti rabbit 647 (ThermoFisher Scientific, 1:500); goat anti-rat 568 (ThermoFisher Scientific, 1:500), donkey anti rabbit 568 (ThermoFisher Scientific, 1:500), donkey anti rat 647 (ThermoFisher Scientific, 1:500). DAPI (2 μg ml^−1^, Sigma-Aldrich) was used to stain nuclei.

### Immunofluorescence on sections

For immunofluorescence on tissue sections, gastruloids were washed in PBS and fixed O/N in 4% PFA, at 4 °C on an orbital shaker. The following day, samples were washed in PBS and embedded in HistoGel (ThermoFisher). HistoGel blocks were then processed with a Tissue-Tek VIP 6 AI Vacuum Infiltration Processor (Sakura) and embedded in paraffin. 4 µm paraffin sections were obtained with a Hyrax M25 microtome (Zeiss). Slides were processed through de-waxing and antigen retrieval in citrate buffer at pH6.0 (using an heat-induced epitope retrieval PT module, ThermoFisher Scientific) before proceeding with immunostaining. Sections were then blocked and permeabilized for 30 min in 1% BSA, 0.2% Triton X-100 in PBS and blocked for 30 min in 10% goat or donkey serum (Gibco) in PBS at RT. Primary antibodies were incubated O/N at 4 °C in PBS, 1.5% donkey or goat serum. On the following day, slices were washed twice in 1% BSA, 0.2% Triton X-100 in PBS and incubated with secondary antibodies at RT for 45 min. Finally, slices were washed twice in 0.2% Triton X-100 in PBS and mounted with Fluoromount-G (SouthernBiotech). Pictures were acquired with an upright Leica DM5500 scanning microscope equipped with a CCD DFC 3000 black and white camera. The following primary and secondary antibodies were used: rabbit anti-CD41 (ThermoFisherScientific, PA5-79526, 1:200); donkey anti rabbit 647 (ThermoFisher Scientific, 1:500). DAPI (2 μg ml^−1^, Sigma-Aldrich) was used to stain nuclei.

### Analysis of single cell-RNAseq data

Single cell analysis was performed with Seurat v3.1 on the dataset described in^9^ and available at NCBI GEO (http://www.ncbi.nlm.nih.gov/geo/) under the accession number GSE158999, using codes described in^9^ and available at (https://github.com/nbroguiere/Cardiac_Gastruloids), focusing on a close-up view of clusters relevant for this paper (as shown in Fig. 1).

### RNA extraction and qRT-PCR

RNA extraction was performed using the RNeasy Micro kit (Qiagen), according to manufacturer’s instructions. RNA was quantified with a Nanodrop (ND-1000). 1 μg of RNA was reverse-transcribed using the iScript cDNA Supermix kit (Biorad). 1.5 μl of 1:5 diluted cDNA was used per reaction, in a total volume of 10 μl. 384 plates were loaded with an automated liquid handling system (Hamilton Microlab Star). qPCR was performed with a 7900HT Fast PCR machine (Applied Biosystems), using Power SYBR Green PCR Master Mix (Applied Biosystems), with an annealing temperature of 60 °C. β-actin expression was used to normalize data. Relative fold expression was calculated with the 2 − ΔΔCT method. 500 nM of primers were used. Primer sequences are shown in Table S1.

### Flow cytometry analysis and cell sorting

For flow cytometry analysis and cell sorting, gastruloids were washed once in PBS and digested in 4 mg ml^−1^ dispase I (Roche), 3 mg ml^−1^ collagenase IV (Gibco) and 100 μg ml^−1^ DNase I (Roche) in PBS (2 digestion cycles at 37 °C, 4 min each, each followed by mechanical dissociation through gentle pipetting). Digestion was blocked with 10% FBS. Samples were then centrifuged, resuspended in sorting buffer (PBS, 10% FBS, 1 mM EDTA, 1% P/S) and incubated for 45 min with antibodies and 10 min with DAPI (2 μg ml^−1^, Sigma-Aldrich), always on ice. Unstained, fluorescent minus one (FMO) and single-color samples were used as controls. Samples were analyzed with a BD LSR Fortessa flow cytometer. Cell sorting was performed with the Aria Fusion cell sorter (BD Bioscience). The following antibodies and dilutions were used: CD34 eFluor660 Mouse Rat IgG2a kappa (eBioscience 50-0341-82, cloneRAM34) 1:80; Sca1 BV711 Mouse Rat IgG2a kappa (Biolegend 108131, clone D7) 1:80; c-Kit PE-Cy5 Mouse Rat IgG2b kappa (eBioscience 15-1171-82 , clone2B8) 1:640; CD31 PE Mouse Rat IgG2a kappa (BD Pharmingen 561073, clone MEC13.3) 1:1280; CD48 BV421 Mouse Amerian Hamster IgG (Biolegend 103427, clone HM48-1) 1:640; CD41 BV605 Mouse Rat IgG1 kappa (BD Pharmingen 747728, clone MWReg30) 1:80; TER119 APC-Cy7 Mouse Rat IgG2b kappa (Biolegend 116223, clone TER-119) 1:160; CD93 PE-Cy7 Mouse Rat IgG2b kappa (Biolegend 136506, clone AA4.1) 1:640; CD45 AF700 Mouse Rat IgG2b kappa (Biolegend 103128, clone 30-F11) 1:30.

### Colony-forming unit assay

To test their colony formation capacity (CFC), cells with presumptive hemogenic capacity were sorted from 168 h gastruloids based on surface marker expression. Sorted cells were added at serial dilutions to 1.5 ml of MethoCult (Stem Cell Technologies, H4434). The mixture of MethoCult and cells was vortexed and transferred with a blunt end needle syringe (Stem Cell Technologies, 28110) into meniscus-free SmartDish plates (6-well-plate, Stem Cell Technologies, 27370). To allow constant humidification, the 6-well plates were placed in a large 150 mm square dish (Corning, CLS431111) containing small 3.5 × 10 mm tissue culture dishes filled with water. The cultures were incubated at 37 °C, 5% CO2 for 14 days and the CFC content was automatedly imaged after 7 and 14 days by using the StemVision imaging system (Stem Cell Technologies, Version 2.0.1.0). Additionally, higher-resolution images of colonies were acquired with a PALM Microbeam microscope (Zeiss) equipped with an AxioCamICc1 color camera and 5 × and 10 × objectives.

### Transplantation and Spleen assay

For transplantation, *Gata6-Venus* (CD45.2) Gastruloids at 168 h were dissociated as described for flow cytometry analysis. 10 weeks old, female, Bl6, CD45.1 recipient mice were lethally irradiated 24 h before transplantation with a split dose of 2 × 425 cGy. 5 × 10^6^ dissociated gastruloid cells were injected in the mice tail vein together with 2 × 10^4^ competitor bone marrow cells isolated from Bl6, CD45.1/2 mice, in a total volume of 200 µl. Control mice received just 2 × 10^4^ competitor bone marrow cells isolated from Bl6, CD45.1/2 mice, in a total volume of 200 µl. Mice were euthanized 11 days after transplantation, and spleens were collected for analysis. One control mouse did not survive until the end of the experiment. All other mice were included in the analysis. No randomization or blinding was applied. Whole spleen images were acquired with a Leica MZ16 1FA stereomicroscope equipped with Leica CLS 150X illumination and DFC480 color camera. All mice were purchased from Charles River Laboratories International and maintained at EPFL conventional animal facility, in microisolator cages, provided with food and water ad libitum. Animal experiments were performed in compliance with the Swiss law after approval from the local and federal authorities (Affaires Vétérinaires de l’Etat de Vaud, license VD2242.2). Reporting in the manuscript follows the recommendations in the ARRIVE guidelines.

### Statistics

All data in graphs are represented as mean ± SD. Statistical analysis between two columns was performed using two-tailed unpaired Student’s t test. Statistical significance was calculated using the Graphpad Prism software, that was also used to generate all graphs.

*P < 0.05; **P < 0.01; ***P < 0.001; confidence intervals 95%; alpha level 0.05.

## Supplementary Information

Supplementary Information.

## Data Availability

The scRNAseq dataset analysed for the current study is described in^9^ and publicly available at NCBI GEO (https://www.ncbi.nlm.nih.gov/geo/) under the accession number GEO: GSE158999. All flow cytometry files and immunofluorescence images are available at the following link: https://doi.org/10.5281/zenodo.6685676. All the other data generated or analysed during this study are included in this published article (and its Supplementary Information files).
