# Supplementary material for: Gastruloids as in vitro models of embryonic blood development with spatial and temporal resolution
Source: Sci Rep. 2022 Aug 4;12:13380. doi: 10.1038/s41598-022-17265-1 (PMC9352713; doi:10.1038/s41598-022-17265-1)
Supplement: Supplementary file 1 — Supplementary Information. [file 41598_2022_17265_MOESM1_ESM.pdf]

Supplementary Figures

Gastruloids as in vitro models of embryonic blood development with spatial and temporal resolution

Giuliana Rossi[1, 3, 4,*], Sonja Giger[1, 4], Tania Hübscher[1], Matthias P. Lutolf[1, 2, 3*]

[1] Laboratory of Stem Cell Bioengineering, Institute of Bioengineering, School of Life Sciences and School of Engineering, École Polytechnique Fédérale de Lausanne (EPFL), Vaud, 1015 Lausanne, Switzerland.

[2] Institute of Chemical Sciences and Engineering, School of Basic Science, École Polytechnique Fédérale de Lausanne (EPFL), Vaud, 1015 Lausanne, Switzerland.

[3] Present address: Roche Institute for Translational Bioengineering (ITB), Roche Pharma Research and Early Development, Roche Innovation Center Basel, Basel, Switzerland.

[4] These authors contributed equally: Giuliana Rossi and Sonja Giger.

*email: giuliana.rossi@roche.com; matthias.lutolf@epfl.ch

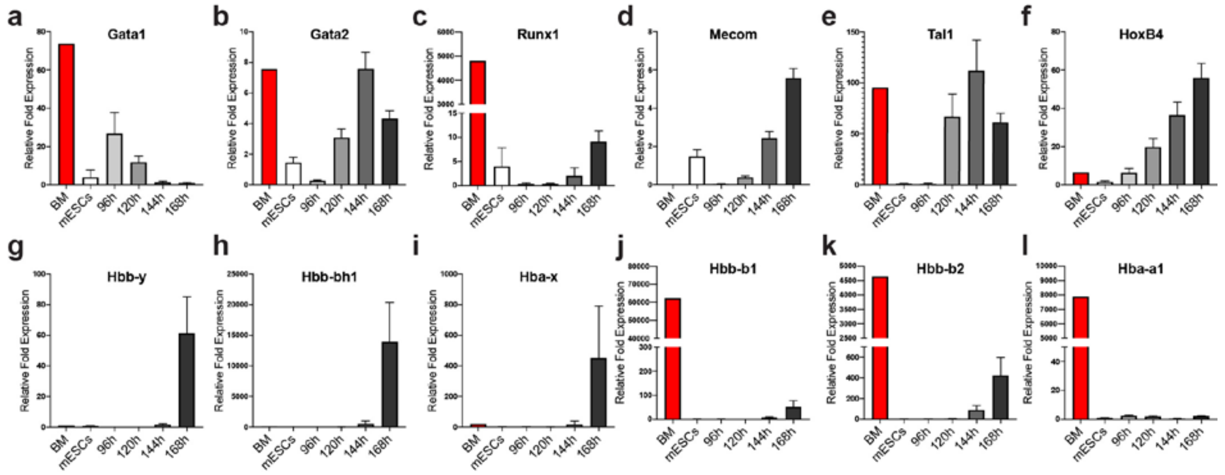

Supplementary Figure 1. Gastruloids express markers of early blood development. (a-f) , qRT-PCR showing the expression of genes associated with hematopoietic development in *Sox1-GFP::Brachyury-mCherry* gastruloids from 96 h to 168 h. **(g-l)**, qRT-PCR showing the expression of embryonic (**g-i**) and adult (**j-l**) hemoglobin genes in *Sox1-GFP::Brachyury-mCherry* gastruloids from 96 h to 168 h. BM, bone marrow. The relative fold expression was calculated as the ratio of the target gene expression in each sample over a mESC sample. Data are expressed as arithmetic mean ± SD, n=4 replicates. *Related to Figure 1.*

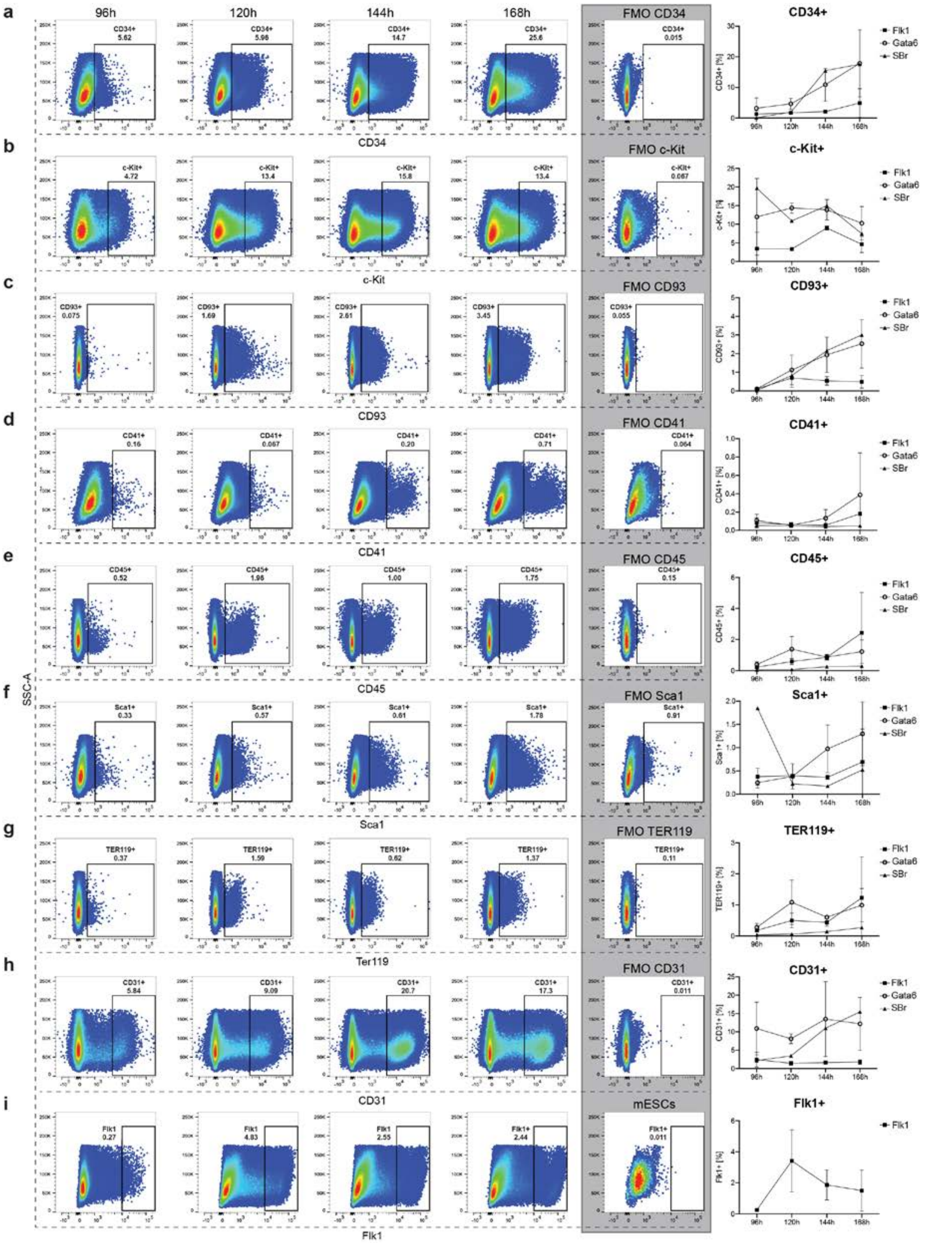

Supplementary Figure 2. Characterization of hematopoietic surface markers in gastruloids. (a-i), Representative flow cytometry plots and relative quantification showing CD34 (**a**), cKit (**b**), CD93 (**c**), CD41 (**d**), CD45 (**e**), Sca1 (**f**), Ter119 (**g**), CD31 (**h**), and Flk1 (**i**) expression in gastruloids from 96 h to 168 h as well as the relative FMO controls (shaded in grey). *Flk1-GFP* (Flk1) (n = 2 replicates), *Gata6-Venus* (Gata6) (n = 2 replicates) and *Sox1-GFP::Brachyury-mCherry* (SBr) (n = 1 replicate). Data are represented as mean ± SD. *Related to Figure 2.*

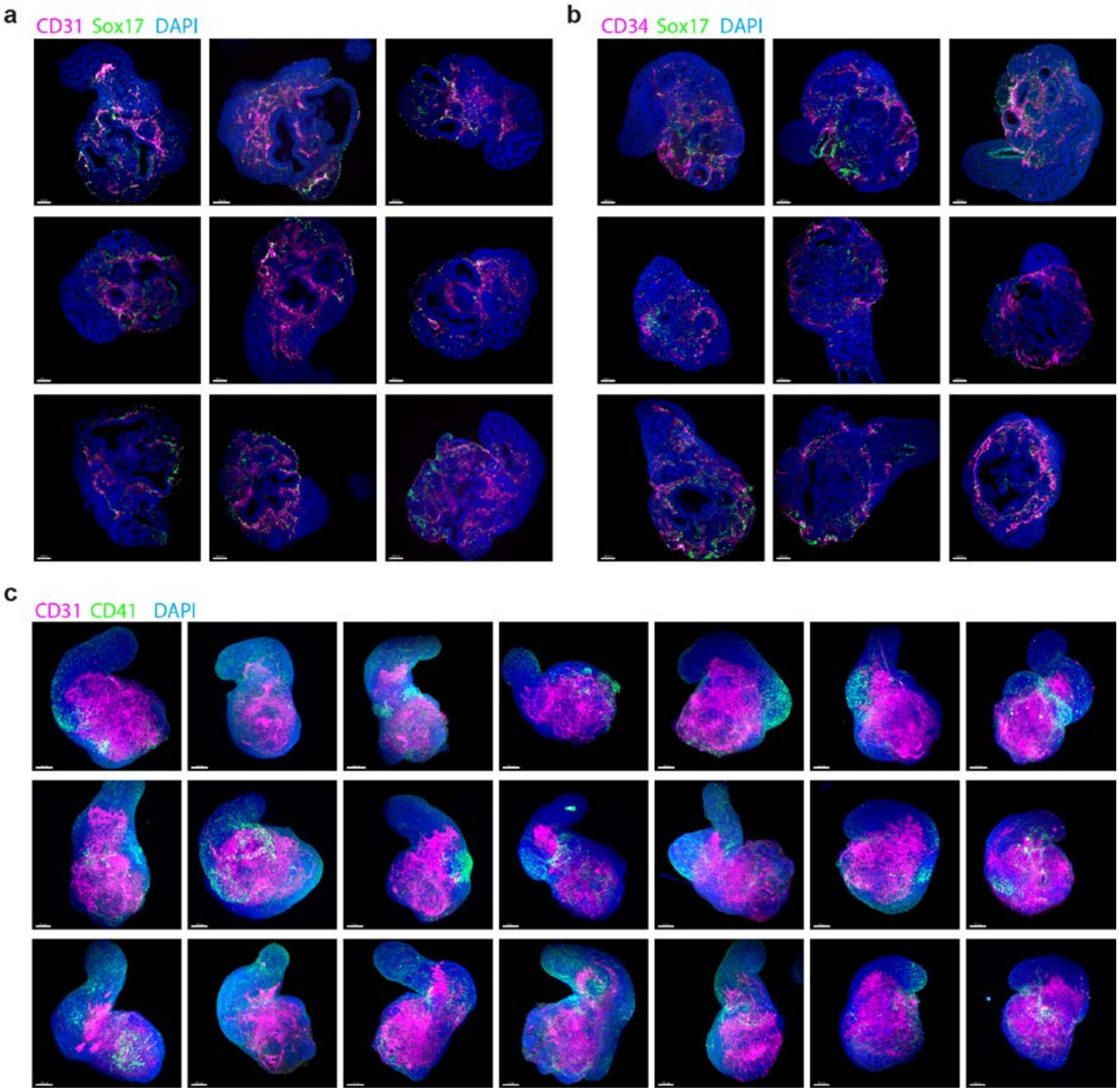

Supplementary Figure 3. Emergence of blood progenitors in gastruloids. (a), Representative collection of light-sheet images of *Gata6-Venus* gastruloids at 168 h showing the co-expression of CD31 and Sox17 in single Z-planes. **(b)**, Representative collection of light-sheet images of *Gata6-Venus* gastruloids at 168 h showing the co-expression of CD34 and Sox17 in single Z-planes. **(c),** Representative collection of light-sheet images of *Gata6-Venus* gastruloids at 168 h showing $CD41^+$ clusters arising in close proximity to the $CD31^+$ vascular network. Scale bars, 150µm. *Related to Figure 3.*

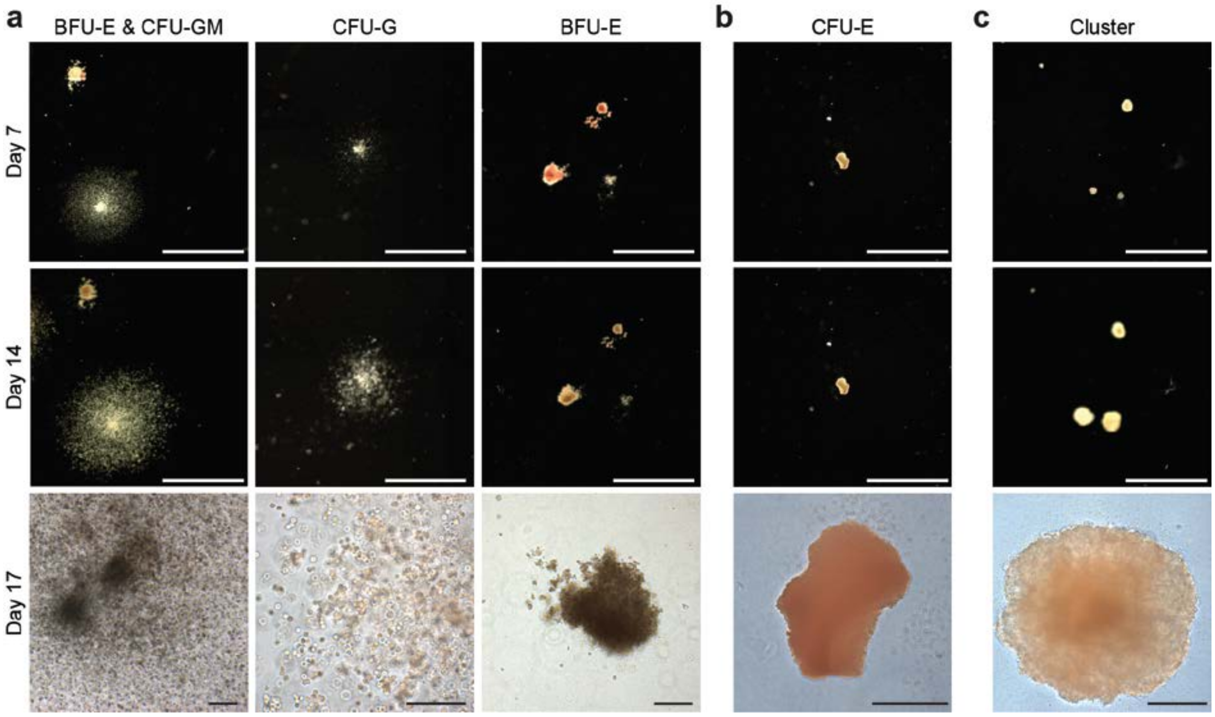

Supplementary Figure 4. Multilineage clonogenic potential of gastruloid-derived blood progenitors. (a), Representative images of BFU-E, CFU-GM and CFU-G colonies derived from cKit$^+$/CD34$^+$/TER119$^-$/CD41$^+$ cells. **(b)**, Representative images of CFU-E colonies derived from the Not(cKit$^+$/CD34$^+$)/TER119$^+$/CD41$^-$ population. We refer to the remaining cells after gating for cKit$^+$/CD34$^+$ cells as Not(cKit$^+$/CD34$^+$). Notably, the Not(cKit$^+$/CD34$^+$) population still contains single positive cells for cKit and CD34. **(c)**, Representative images of cluster formation of *Gata6-Venus* mESCs. All cell populations were cultured in Methocult and imaged after 7, 14, and 17 days.
BFU-E, burst-forming-unit-erythroid; CFU-GM, colony forming unit-granulocyte, macrophage; CFU-E, colony forming unit-erythroid. Scale bars 200µm. *Related to Figure 4.*

Supplementary Tables

Gene:		Primer sequence
β-actin	FOR	CTGTCGAGTCGCGTCCACC
	REV	CGCAGCGATATCGTCATCCA
Gata1	FOR	GTGGCTGAATCCTCTGCATCA
	REV	TAAGGTGAGCCCCCAGGAAT
Gata2	FOR	GCCGGGAGTGTGTCAACTG
	REV	AGGTGGTGGTTGTCGTCTGA
Mecom	FOR	AATAAATCCGAAACGCGTGGT
	REV	CCTACATCTGGTTGACTGGCA
Runx1	FOR	AGGCAGGACGAATCACACTG
	REV	CTCGTGCTGGCATCTCTCAT
Tal1	FOR	CACTAGGCAGTGGGTTCTTTG
	REV	GGTGTGAGGACCATCAGAAATCT
Hoxb4	FOR	AAAGAGCCCGTCGTCTACC
	REV	AGTGTAGGCGGTCCGAGAG
Hbb-bh1	FOR	GGAAACCCCCGGATTAGAGC
	REV	CTGGGGTGAATTCCTTGGCA
Hbb-b1	FOR	TGTCTCTTGCCTGTGGGGAA
	REV	GAAATCCTTGCCCAGGTGGT
Hbb-b2	FOR	TGAAGGCCCATGGCAAAAAG
	REV	CGATCGCATTGCCTAGGAGC
Hba-a1	FOR	CTGAAGCCCTGGAAAGGATGT
	REV	AGAGCCGTGGCTTACATCAAA
Hba-x	FOR	ATCAGGCCAGTCTTGAGTGC
	REV	GGAGCTTGAAGTTGACCGGA
Hbb-y	FOR	TCTGCCATAATGGGCAACCC
	REV	TGCCGAAGTGACTAGCCAAA

Supplementary Table 1. List of primers used for qRT-PCR
